# Improving the energy resolution of bent crystal X-ray spectrometers with position-sensitive detectors

**DOI:** 10.1107/S1600577514011163

**Published:** 2014-06-12

**Authors:** Ari-Pekka Honkanen, Roberto Verbeni, Laura Simonelli, Marco Moretti Sala, Ali Al-Zein, Michael Krisch, Giulio Monaco, Simo Huotari

**Affiliations:** a Department of Physics, PO Box 64, FI-00014 Helsinki, Finland; b European Synchrotron Radiation Facility, BP 220, F-38043 Grenoble Cedex, France; c CELLS – ALBA, Carretera, BP 1413, de Cerdanyola del Vallès a Sant Cugat del Vallès, Km 3.3, 08290 Cerdanyola del Vallès, Barcelona, Spain; d Physics Department, University of Trento, Via Sommarive 14, 38123 Povo (TN), Italy

**Keywords:** X-ray spectrometers, position-sensitive detectors, bent analyser crystals

## Abstract

Wavelength-dispersive high-resolution X-ray spectrometers often employ elastically bent crystals for the wavelength analysis. In a preceding paper [Honkanen *et al.* (2014). *J. Synchrotron Rad.* **21**, 104–110] a theory for quantifying the internal stress of a macroscopically large spherically curved analyser crystal was presented. Here the theory is applied to compensate for the corresponding decrease of the energy resolution. The technique is demonstrated with a Johann-type spectrometer using a spherically bent Si(660) analyser in near-backscattering geometry, where an improvement in the energy resolution from 1.0 eV down to 0.5 eV at 9.7 keV incident photon energy was observed.

## Abstract 2

A new measurement technique for X-ray spectrometers equipped with a position-sensitive detector is introduced. It is based on the computational compensation of the effects of internal stress of curved analyser crystals to improve the energy resolution in the measurements of point-like samples with no loss of intensity.

## 1. Introduction   

X-ray emission spectroscopy (XES) and inelastic X-ray scattering (IXS) spectroscopies in their various incarnations (Cooper, 2007 ▸; Schülke, 2007 ▸; Ament *et al.*, 2011 ▸) are vividly expanding methods for studying the local electronic and real-space structures of materials in various phases and conditions. Different applications require different techniques characterized, for example, by their energy resolution. Applications that are more relaxed when it comes to energy resolution include X-ray-Raman-scattering-based EXAFS (Huotari *et al.*, 2012 ▸; Hiraoka *et al.*, 2013 ▸) and Compton-scattering spectroscopy (Cooper, 2007 ▸) (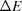 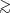 a few eV). Resonant X-ray emission spectroscopy (de Groot & Kotani, 2008 ▸) and X-ray-Raman-scattering-based XANES (Gordon *et al.*, 2008 ▸) would profit from an energy resolution of 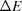 ≃ 300 meV. Resonant IXS, for example at the *K*, *L* and *M* edges of 3*d*, 4*d* and 5*d* transition metals, with 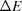 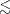 100 meV is nowadays a powerful tool for studying collective magnetic and orbital excitations (Hill *et al.*, 2005 ▸; Schlappa *et al.*, 2012 ▸).

The instruments for the above-mentioned studies are often based on wavelength analysis by diffractive optics. In the soft X-ray IXS range reflective diffraction gratings are used, while in the regime of X-ray wavelengths of the order of 1 Å the typical optics is based on Bragg reflections of single crystals. Different curved-optics designs have been employed throughout the history of X-ray spectroscopy, such as the Johann, Johansson and von Hamos designs for reflection geometry, and Laue or Cauchois designs for transmission geometry. Johann-type Bragg optics is often used with crystal curvature radii of 1–2 m (Verbeni *et al.*, 2009 ▸). The fundamental concept in the Johann optics is to have a crystal surface taking a shape that matches the requirement that all X-rays from the sample impinging on the crystal have almost the same angle of incidence. This angle of incidence transforms to the corresponding energy of the Bragg reflection *via* Bragg’s law. Simultaneously, but only approximately in the Johann geometry, another aim is to have the crystal surface matching the focusing condition to form a real-space image of the footprint of the sample (Huotari *et al.*, 2011 ▸). However, elastic bending causes internal stress that leads to strain deformations of the perfect crystal lattice. These deformations increase the bandwidth of the crystal, simultaneously increasing also the integrated reflectivity which is desirable in applications where sub-eV energy resolution (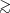
100 meV) is required.

The theoretical framework of the diffraction properties of bent analyser crystals is in principle known from the independent approaches of Takagi and Taupin (Takagi, 1962 ▸, 1969 ▸; Taupin, 1964 ▸). The deformed crystal can also be assumed to consist of small lamellae, the orientation and displacement of which can vary according to a computed strain field (Erola *et al.*, 1990 ▸; del Rio *et al.*, 2004 ▸). Many of the previous approaches have considered the effect of a strain field that depends on the thickness of the crystal only, neglecting the lateral dimension of the crystal. We have shown (Honkanen *et al.*, 2014 ▸) that by taking into account the macroscopic surface area of the crystal, while assuming the wafer to be thin, a component of the strain field that causes an *angular* strain could be isolated. The results of the model were in good agreement with experimental resolution functions obtained with spherically bent circular Si(660) wafers in near-backscattering geometry (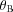 ≃ 89°), with a curvature radius of 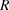 = 1 m.

In this article, we take a step further and show that the angular stress can be visualized to have an almost radial dispersion over the analyser crystal surface and that it can be experimentally verified in an off-focusing geometry. Most importantly, we show that the dispersion on a position-sensitive detector can be used in a novel way to compensate for this angular-stress-caused dispersion and the energy resolution of such a crystal analyser can be improved by this new detection scheme. We demonstrate this in the case of a Si(660) wafer under the conditions mentioned above. An increase of the energy resolution by a factor two was observed.

The article is arranged as follows. After this introductory section, the theory is shortly represented in §2. Experimental verification and results are shown in the following sections.

## 2. Theory of compensation   

**Figure 1:**
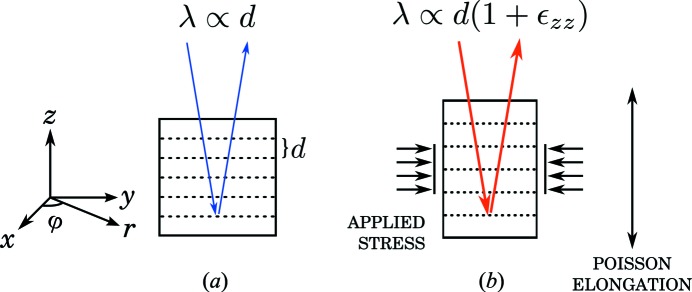
(*a*) The wavelength λ of the diffracted X-rays is proportional to the separation of the crystal planes *d* as described by Bragg’s law. (*b*) Applying stress to the sides of the crystal causes the separation of the crystal planes to change from *d* to 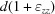, where 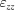 is the strain owing to Poisson elongation. Supposing the stress is uniform and in the limits of linear elasticity, λ is expected to shift along with the separation of the crystal planes by a relative amount of 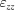. The Cartesian and cylindrical coordinate systems used in this paper are presented on the left.

Consider an unstrained cubic crystal whose top surface normal is along the *z*-direction, as depicted in Fig. 1(*a*) ▸. The wavelength λ of the diffracted radiation is proportional to the separation *d* of the crystal planes. Now, suppose we apply stress to the sides of the cubic crystal as in Fig. 1(*b*) ▸. Owing to the Poisson effect, the compression of the crystal in one or two directions causes it to elongate along the free direction(s). If the applied stress is uniform, the Poisson elongation is constant inside the crystal. The change in the separation of the reflective planes can be taken into account in Bragg’s law by replacing 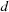 by 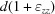, where 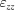 is the Poisson elongation normal to the reflecting crystal planes. Thus it follows that the relative change in energy of the reflected photons due to elongation up to the first order is 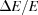 = 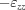 for small 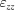.

In reality the X-ray reflectivity of a crystal is a more complicated phenomenon than described above. However, since the reflectivity is based on the local fulfilment of a condition similar to Bragg’s law, our deduction can be extended to real crystals. Suppose that the reflectivity at some arbitrary point of the crystal for the photon energy *E* is 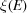, when 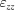 = 0. If we now introduce a strain component 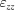, that is constant along the beam path, it causes the reflectivity curve to shift as 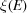 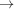 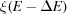, up to first order in 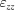. Now if 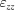 varies as a function of *x* and *y*, it degrades the energy resolution of the analyser as a whole owing to a summation of these energy-shifted curves, even if 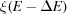 is locally unaffected.

**Figure 2:**
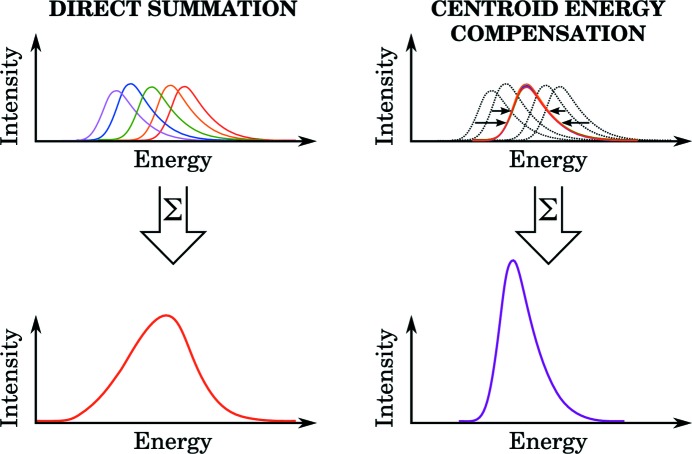
The principle of sub-analyser compensation. Before summing up all spectra measured by different regions of a spherical analyser, the peak (or centroid) energies of the spectra are calculated and the spectra are shifted accordingly.

The idea introduced in the previous paragraph is supported by experiments. The FWHM of the resolution curve of a bent analyser is found to improve when its active surface area is made smaller (Verbeni *et al.*, 2005 ▸, 2009 ▸; Honkanen *et al.*, 2014 ▸). The obvious drawback of this approach is that the solid angle of the analyser is reduced. However, as we will show in this article, the local energy resolution of 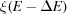 can be recovered if different parts of the illuminated area of the analyser are spatially resolved with a two-dimensional detector. A concept to perform this task *via* an off-focus imaging technique is introduced in §3. The fundamental idea is that the surface of the analyser can be divided into multiple sub-analysers that are small enough so that their reflectivity curves are approximately 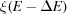 with 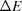 varying from a sub-analyser to another. An energy calibration can be derived by scanning the energy across the elastic line to obtain the different 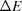 values and then used to compensate for these energy shifts. Consequently, the energy resolution of the whole analyser is improved without reducing the diffracted intensity. This process is depicted in Fig. 2 ▸. It should be noted that the benefits of spatial resolving are not limited only to the improvement of the energy resolution, but it can also be applied to enhance, for example, the momentum transfer resolution of the measurement.

### 2.1. The case of spherically bent analysers   

The reflectivity curves for distorted crystals can be calculated, for example, from the Takagi–Taupin theory. In the case of spherically bent crystals the solutions are usually obtained assuming a depth-dependent strain field, which is the first-order approximation neglecting the components of strain parallel to the crystal surface. However, despite being small their contribution to the X-ray reflectivity of the crystal analyser cannot be neglected. As shown by Honkanen *et al.* (2014 ▸), the compression in the angular direction in commonly used spherical analysers can cause additional strains of the order of 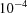. Since this is comparable with the contribution from a depth-dependent strain field, it cannot be neglected. The shift in the centroid energy 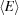 of the local reflectivity curve as a function of the position on the crystal surface is given by (Honkanen *et al.*, 2014 ▸)
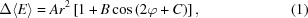
where *r* is the distance from the centre of the analyser and φ is the angular coordinate (see the coordinate system in Fig. 1 ▸). The constants are
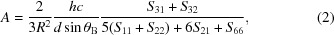


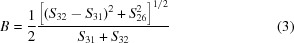
and
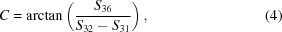
where *R* is the bending radius of the analyser, *h* is Planck’s constant, *c* is the speed of light in a vacuum, *d* is the separation of the undistorted Bragg planes of the reflection, 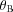 is the Bragg angle and 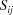 are the Cartesian components of the compliance matrix of the crystal, formed from the compliance tensor following the Voigt notation. The Cartesian and cylindrical systems are related as shown in Fig. 1 ▸.

## 3. Off-focus imaging   

**Figure 3:**
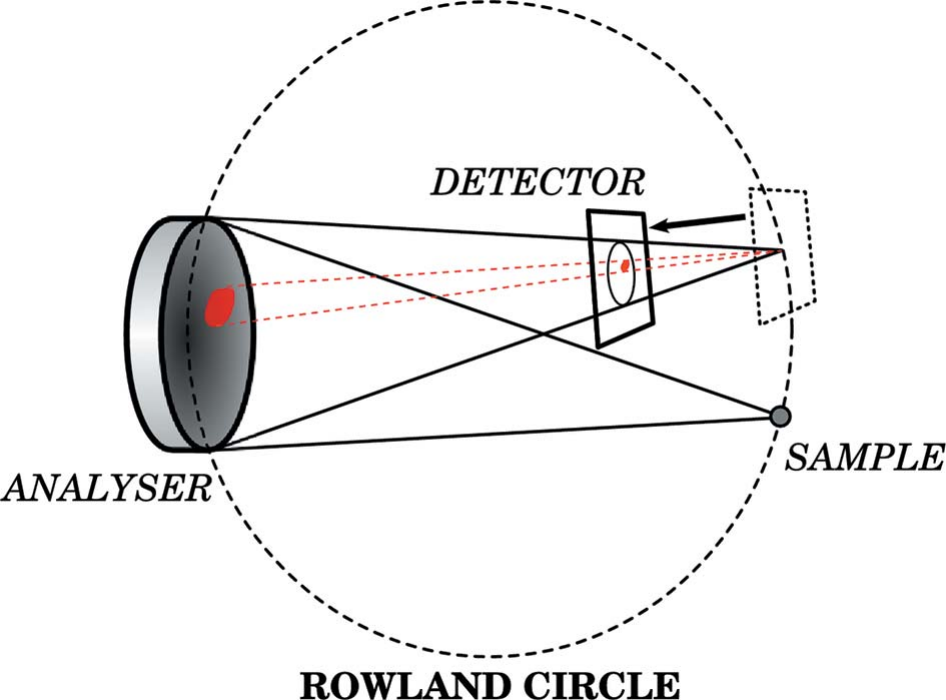
Off-focus scan geometry. The position-sensitive detector is moved off the Rowland circle towards the analyser in order to resolve the position dependence of the reflectivity. The red lines illustrate how the surface of the analyser is resolved on the detector.

**Figure 4:**
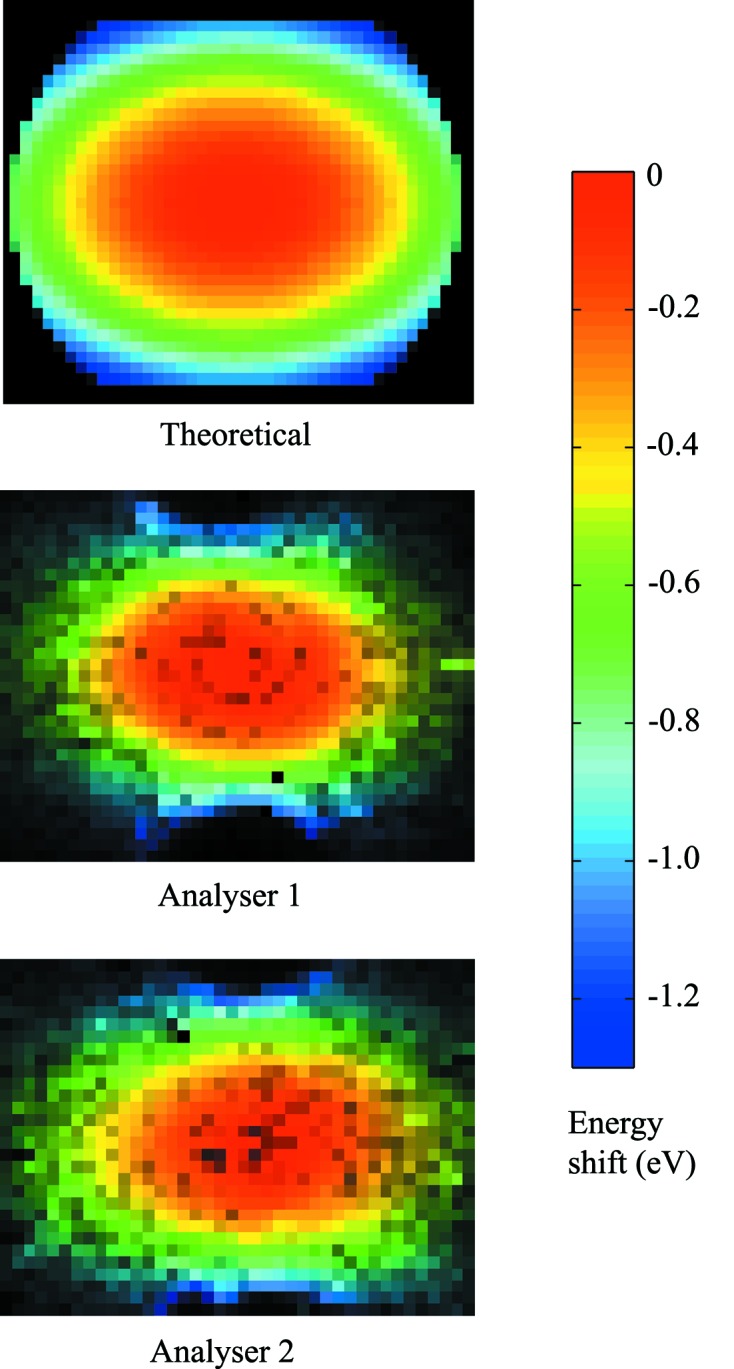
Predicted and measured centroid energy distributions on the surface of a Si(660) analyser with 100 mm diameter and 1 m bending radius at a Bragg angle of 88.7° measured with a position-sensitive detector in off-focus geometry. 10 mm stripes were cut off the top and the bottom of the analysers leaving a maximum vertical size of 80 mm. The detector was displaced 20 mm inward to the Rowland circle, leading to a focus of 2 mm (40 pixels) in horizontal size. The colour corresponds to the shift in energy, whereas the brightness indicates the collected intensity. The method used to image the experimental energy-intensity map is described in §3.

Consider a measurement set-up that consists of a position-sensitive detector (PSD), a spherically bent analyser crystal and a sample to be studied. Conventionally in Johann geometry (Johann, 1931 ▸) all three are placed on the so-called Rowland circle whose radius is half the bending radius of the analyser. Suppose the PSD is brought inside the circle (see Fig. 3 ▸). Now the X-rays reflected by the analyser do not focus on a single spot on the detector, but there is a one-to-one correspondence between a detector pixel recording an X-ray photon and an analyser surface element from which it originated. Each detector pixel thus records a signal reflected by a specific part of the analyser, and one is effectively measuring an image of the analyser surface diffraction properties. This allows us to study how different parts of the analyser reflect X-rays. Results from such an off-focus imaging procedure are presented in Fig. 4 ▸.

## 4. Experimental verification   

In this section we apply the compensation method described in §2 to simulated and measured off-focus spectra. The experimental set-up and the simulation procedure will be explained in detail in the following.

### 4.1. Experimental   

The measured quantity in IXS is the double differential cross section, which can be written in terms of the dynamic structure factor 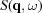,
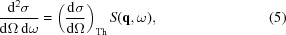
where 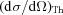 is the Thomson scattering cross section. Here and below, 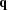 and 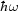 are the momentum and energy transferred to the electron system, respectively. 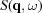 is
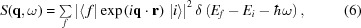
where 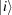 and 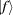 are the initial and final states, respectively, and 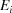 and 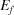 their corresponding energies.

We use as an example the 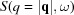 spectra of 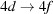 transitions of the La^3+^ ion in LaPO_4_, similar to as reported by Gordon *et al.* (2008 ▸). The measurements were conducted at the European Synchrotron Radiation Facility (ESRF) beamline ID20 using the five-analyser high-resolution spectrometer. The sample was a powder of LaPO_4_ nanoparticles (Huotari *et al.*, 2014 ▸). The incident photon energy 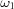 was scanned between 9.69 and 9.8 keV to span the zero-loss peak to determine the energy resolution, as well as the energy-transfer range 98–107 eV where non-dipolar high-order multipole transitions (Gordon *et al.*, 2008 ▸) of La^3+^ can be observed. For the analysis here, two spherically bent Si(660) analysers with bending radii of 1 m were used, collecting scattered radiation at angles 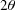 = 120–140°. The momentum transfer was ∼8 Å^−1^. The experimental results are compared with results from an atomic multiplet code (Cowan, 1981 ▸) that was used to calculate the required transitions for a La^3+^ ion. This approach has been shown to work well for the spectra for 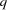 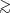 7 Å (Gordon *et al.*, 2008 ▸).

The spectra were measured with the detector moved 20 mm inside the Rowland circle. The focus on the detector was 2 mm (40 pixels) in diameter, meaning that an individual pixel saw a 2.5 mm × 2.5 mm portion of the analyser surface. The worsening of the energy resolution owing to the angular compression for an area of that size is 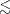
30 meV, thus allowing us to use the previously described dispersion compensation method.

### 4.2. Simulation   

The IXS spectrum was simulated as follows. The intensities of La^3+^ transition peaks relative to each other were obtained using the aforementioned atomic multiplet code. The intrinsic widths of the peaks were assumed to be negligible compared with the resolution of the measurement set-up, *i.e.*

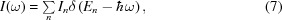
where 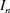 and 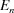 are the intensity and the energy of the *n*th transition, respectively, and 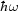 is the energy transferred to the electron system.

The contributions of the depth-dependent strain field of the crystal and the incident bandwidth (FWHM 235 meV) to the shape of the intensity curve were taken into account by convoluting the delta function intensity with the Takagi–Taupin and Gaussian curves, respectively. The Johann error and the contribution source size were negligible (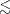
10 meV). The Takagi–Taupin curve for the depth-dependent strain field was calculated according to Gronkowski (1991 ▸). The experimental spectra were normalized to have the same intensity as the theoretical ones. The obtained curve corresponds to a large photon count spectrum measured using an analyser that is free of angular compression.

Since in near-backscattering conditions spherical aberration and Johann error are small, the image on the detector taken off-focus is a scaled image of the analyser surface. Therefore the surface of the analyser can be divided into an equally spaced square grid, where each square represents the area that is seen by an individual pixel of the detector. For an accurate simulation, the pixels were further subdivided onto a finer grid in order to take into account the variation of angular compression to the resolution function of an individual pixel. The shift in the centroid energy was calculated for every sub-grid point using equation (1). The spectrum seen by a pixel was then obtained by translating the previously computed surface area distribution according to the calculated 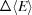 values and summing the shifted curves together. In order to perform a realistic calculation, we generated the simulated spectrum from virtual photon counts with statistical accuracy that matches that of the experimental spectrum. The vertical size of the simulated reflection was 40 pixels and each of the pixels was divided into 625 sub-points. The simulated scans were compensated using the very same routines as used for the measured data.

### 4.3. Results   

**Figure 5:**
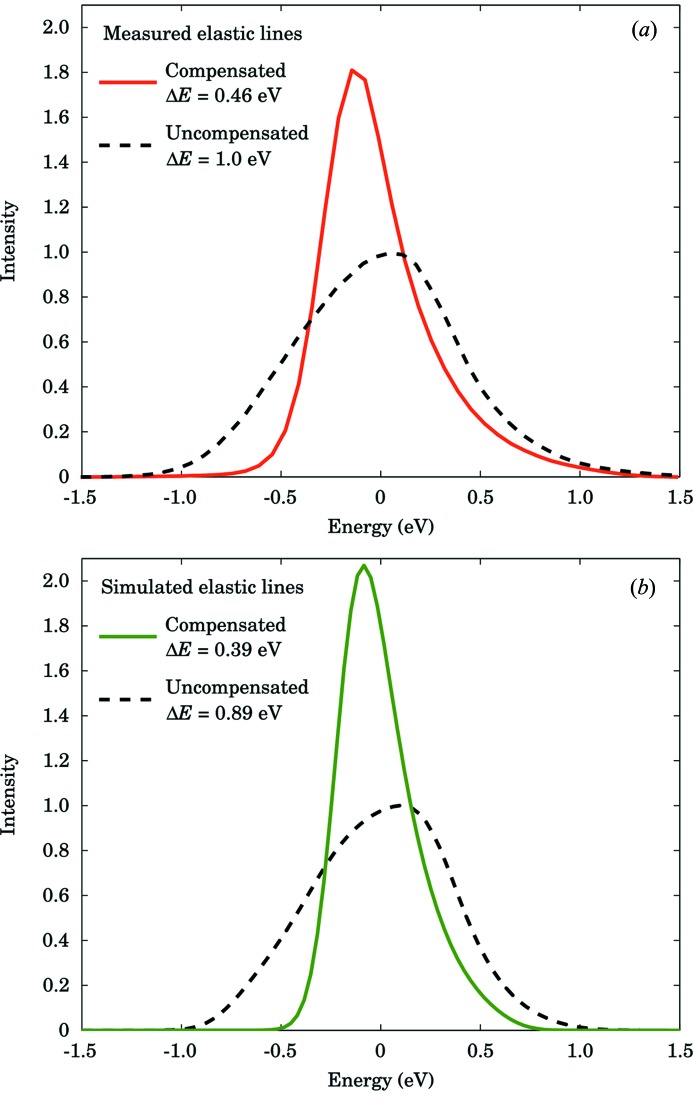
Comparison of (*a*) measured and (*b*) simulated compensated (solid lines) and uncompensated (dashed lines) elastic lines of the Si(660) analyser. For the measured lines the FWHMs are 1.0 eV for the uncompensated line and 0.46 eV for the compensated one. For the simulated lines the FWHMs are 0.89 eV and 0.39 eV, respectively.

The measured quasi-elastic lines of LaPO_4_ that represent the resolution functions are shown in Fig. 5(*a*) ▸. The data are shown as integrated over the full focus, which corresponds to a traditional measurement (*i.e.* with a point detector), and after applying the compensation algorithm explained in the previous sections. We compare the results with a simulation performed as explained in §4.2, in which we assumed an infinitely sharp excitation centred at 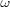 = 0 eV and measured by a theoretical analyser that has an angular-stress distribution as depicted in Fig. 4 ▸. The simulated resolution functions are presented in Fig. 5(*b*) ▸. In the simulation, the FWHM of the resolution function can be improved from 0.89 eV to 0.39 eV. In the experimental result, we demonstrate here an improvement from 0.93 eV down to 0.43 eV, implying that the compensation for the spectrum is practically optimal.

**Figure 6:**
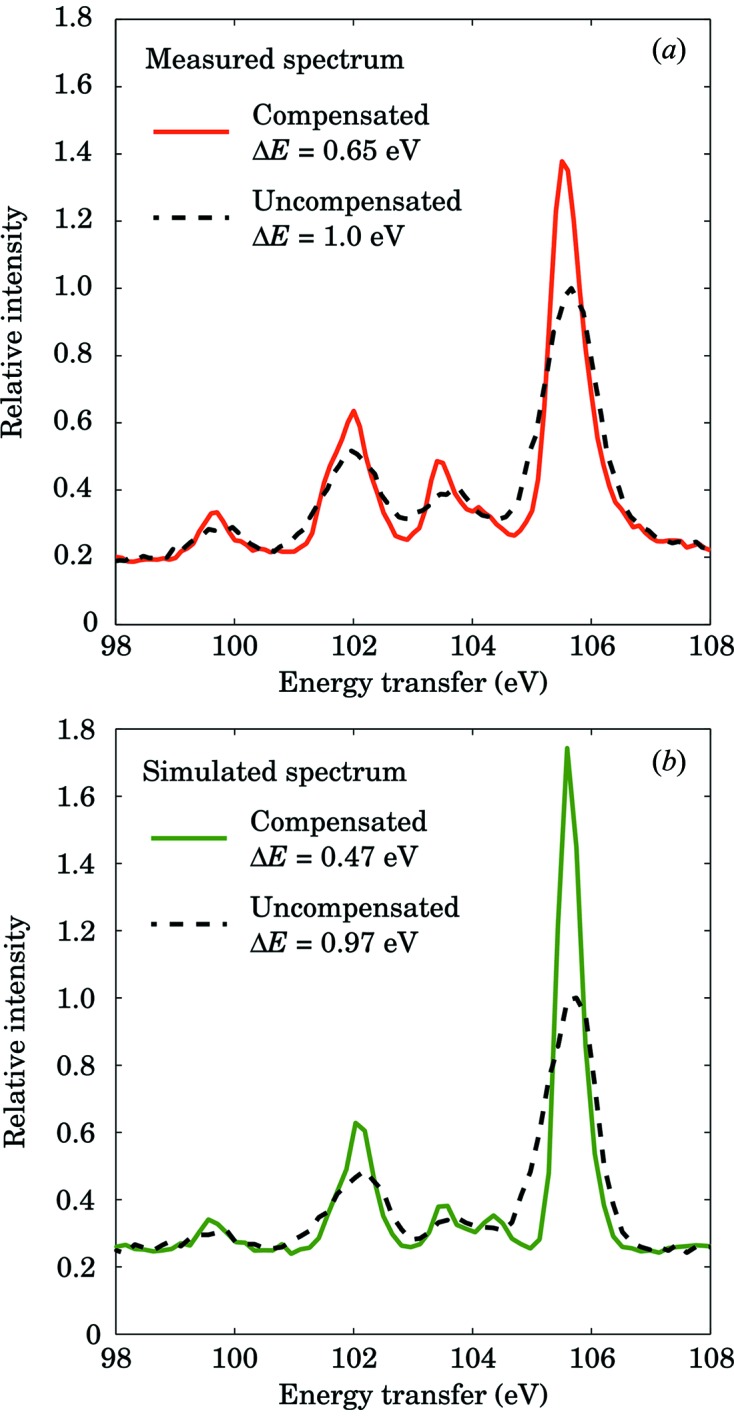
Comparison of compensated (solid lines) and uncompensated (dashed lines) IXS spectra of (*a*) measured and (*b*) simulated La^3+^ 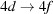 transitions in LaPO_4_. The FWHMs of the 105.5 eV peak for both spectra are presented in the legend.

The measured IXS spectra are shown in Fig. 6(*a*) ▸. The 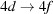 spectrum in La^3+^ at the considered momentum transfer is dominated by non-dipolar transitions in the energy transfer range 100–110 eV. When applied to the measured IXS spectrum, the compensation algorithm improves the resolution, and, for example, the FWHM of the strongest single line at 105.5 eV narrows down from 1.0 eV (without compensation) to 0.65 eV (with compensation) as shown in Fig. 6(*b*) ▸. The change of the line width after compensation is not as large as expected theoretically when assuming excitations to have negligible bandwidth. The remaining finite bandwidth in the experimental result may reflect a real width of the excitation.

The excellent agreement of the simulated and experimental resolution functions shows that the proposed technique performs as anticipated. This also supports the validity of the theory of angular compression (Honkanen *et al.*, 2014 ▸), and shows that it is applicable in the case of a typical bent-crystal X-ray spectrometer. The results demonstrate an improvement of the resolving power up to a factor of two, with no loss of diffracted intensity.

## 5. Conclusions   

In this work we have introduced a new measurement technique for X-ray spectrometers equipped with a position-sensitive detector. The technique is based on the computational compensation of the effects of internal stress of curved analyser crystals to improve the energy resolution in the measurements of point-like samples with no loss of intensity.

A demonstration of the technique is given using both simulated and measured IXS data. The energy resolution of the measured data is improved from 1.0 eV to 0.5 eV. The results help to explain the reflectivity properties of spherically bent crystal analysers and demonstrate how the understanding of these properties can yield new techniques for improving the energy resolution of such diffractive optics.

## References

[bb1] Ament, L. J. P., van Veenendaal, M., Devereaux, T. P., Hill, J. P. & van den Brink, J. (2011). *Rev. Mod. Phys.* **83**, 705.

[bb2] Cooper, M. (2007). *X-ray Compton Scattering.* Oxford University Press.

[bb3] Cowan, R. D. (1981). *The Theory of Atomic Structure and Spectra.* Berkeley: University of California Press.

[bb4] Erola, E., Eteläniemi, V., Suortti, P., Pattison, P. & Thomlinson, W. (1990). *J. Appl. Cryst.* **23**, 35–42.

[bb5] Gordon, R. A., Seidler, G. T., Fister, T. T., Haverkort, M. W., Sawatzky, G. A., Tanaka, A. & Sham, T. K. (2008). *Europhys. Lett.* **81**, 26004.

[bb6] Gronkowski, J. (1991). *Phys. Rep.* **206**, 1–41.

[bb7] Groot, F. de & Kotani, A. (2008). *Core Level Spectroscopy of Solids.* Boca Raton: CRC Press.

[bb8] Hill, J. P., Blumberg, G., Kim, Y.-J., Ellis, D. S., Wakimoto, S., Birgeneau, R. J., Komiya, S., Ando, Y., Liang, B., Greene, R. L., Casa, D. & Gog, T. (2005). *Phys. Rev. Lett.* **100**, 097001. 10.1103/PhysRevLett.100.097001 18352743

[bb9] Hiraoka, N., Fukui, H., Tanida, H., Toyokawa, H., Cai, Y. Q. & Tsuei, K. D. (2013). *J. Synchrotron Rad.* **20**, 266–271. 10.1107/S0909049512048789 23412483

[bb10] Honkanen, A.-P., Verbeni, R., Simonelli, L., Moretti Sala, M., Monaco, G. & Huotari, S. (2014). *J. Synchrotron Rad.* **21**, 104–110. 10.1107/S160057751302242X 24365923

[bb11] Huotari, S., Pylkkänen, T., Soininen, J. A., Kas, J. J., Hämäläinen, K. & Monaco, G. (2012). *J. Synchrotron Rad.* **19**, 106–113. 10.1107/S0909049511039422 22186651

[bb12] Huotari, S., Pylkkänen, T., Verbeni, R., Monaco, G. & Hämäläinen, K. (2011). *Nat. Mater.* **10**, 489–493. 10.1038/nmat3031 21623376

[bb13] Huotari, S., Suljoti, E., Rädl, S. & de Groot, F. M. F. (2014). In preparation.

[bb22] Johann, H. H. (1931). *Z. Phys.* **69**, 185–206.

[bb14] Rio, M. S. del, Alianelli, L., Faenov, A. Y. & Pikuz, T. (2004). *Phys. Scr.* **69**, 297–302.

[bb15] Schlappa, J., Wohlfeld, K., Zhou, K. J., Mourigal, M., Haverkort, M. W., Strocov, V. N., Hozoi, L., Monney, C., Nishimoto, S., Singh, S., Revcolevschi, A., Caux, J. S., Patthey, L., Rønnow, H. M., van den Brink, J. & Schmitt, T. (2012). *Nature (London)*, **485**, 82–85. 10.1038/nature10974 22522933

[bb16] Schülke, W. (2007). *Electron Dynamics Studied by Inelastic X-ray Scattering.* Oxford University Press.

[bb17] Takagi, S. (1962). *Acta Cryst.* **15**, 1311–1312.

[bb18] Takagi, S. (1969). *J. Phys. Soc. Jpn*, **26**, 1239–1253.

[bb19] Taupin, D. (1964). *Bull. Soc. Fr. Mineral. Crystallogr.* **87**, 469–511.

[bb20] Verbeni, R., Kocsis, M., Huotari, S., Krisch, M., Monaco, G., Sette, F. & Vankó, G. (2005). *J. Phys. Chem. Solids*, **66**, 2299–2305.

[bb21] Verbeni, R., Pylkkänen, T., Huotari, S., Simonelli, L., Vankó, G., Martel, K., Henriquet, C. & Monaco, G. (2009). *J. Synchrotron Rad.* **16**, 469–476. 10.1107/S090904950901886X 19535859

